# New Appliances and Things Medical

**Published:** 1895-07-27

**Authors:** 


### NEW APPLIANCES AND THINGS MEDICAL.

[We shall be glad to receive, at our Office, 428, Strand, London, W.C., from the manufacturers, specimens of all new preparations and appliances which may be brought out from time to time.]

### LAWES' DISINFECTANT FLUID AND POWDER.

(Lawes' Chemical Company, 59, Mark Lane, London, E.C.)

From our examination of this new disinfectant we have come to the conclusion that it is a creosote preparation made miscible with water by the addition of an alkaline salt. On the addition of water it forms a milky-white emulsion, and when a drop of the liquid is allowed to run down on to the surface of a clock glass of water it was rapidly taken up, and played over its surface, showing perfect miscibility. On distillation we obtained 6 per cent. of water, 2·56 per cent. of light oils, distilling below 200° C., and 73·4 per cent. of heavy oils, with a carbonised residue of 20 per cent. left in the retort. The total quantity of mineral matter is about 4 per cent., and this is somewhat higher than that usually met with in such preparations. When extracted with soda, 100 cub. cent. of the disinfectant yielded 19 cub. cent. of tar acids, and such a result corresponds to the yield of tar acids, which is obtained from an English country creosote oil made in the Midlands, which is much richer in tar acids than the tar obtained from London oils. This satisfactory result shows that the liquid should have marked disinfectant properties, but the removal of the phenic acid, as stated in the report, and the low percentage of light oils found, points to a fractional distillation having taken place, which has also probably diminished the amount of pyridine bases normally present in such oils. The manufacturers make a point of the preparation being non-poisonous, and although it is customary to regard a phenol-free oil as being non-poisonous it must not be forgotten that the higher homologues which are present in this compound are not absolutely free from danger. The instructions recommend the use of a solution containing a quarter of a pint to three gallons of water for rinsing infected clothes. This strength (1 in 96) is far too weak to be effective, as shown by our own experiments on the disinfectant power of this fluid. Henri Bergé's report of the disinfectant, although claiming for this fluid a high disinfecting power, does not give any details of experiments with specific organisms, and we are hence of the opinion that for satisfactory germicidal action the dose of this disinfectant must be considerably higher. Spraying a room with a solution (1 in 96) is also recommended, and must be condemned as valueless, not only for the above reason, but also because solutions of non-volatile disinfectants give very unsatisfactory results when used in this way by inexperienced hands. In our experiments upon the disinfectant action of this fluid, we tested its action upon the three organisms Staphylococcus pyogenes aureus, Bacillus coli communis, and Spirillum choleræ. Solutions of the disinfectant were made of the following strengths: 1 in 50, 1 in 250, 1 in 500, 1 in 5,000. Five cub. cent. of these solutions were added to 5 cub. cent. of a suspension in sterile distilled water of twenty-four hours old agar agar cultures of the specified organisms. The strength of the solutions actually employed was consequently half the above strength in each case. I.—The 1 in 10,000 solution was found not to kill B. coli in five days, and the Sp. choleræ remained alive in it not less than four hours; but as an uninoculated solution of this strength did not remain sterile, further experiments with it were abandoned. II.—1 in 1,000 solution: Staph. pyog. aureus was alive after 50 hours; B. coli was alive after 5 days; and Sp. chol· after 20 minutes, but dead after 30 minutes' exposure. III.—1 in 500 solution: Staph. pyog. aureus was alive after 50 hours, as also was B. coli; Spir. chol. was dead after 10 minutes.

| IV.—1 in 100 Solution. | Alive. | Dead. |
|---|---|---|
| Staph. pyog. aureus ... | after 4 hours ... | after 5 hours. |
| Bac. coli. ... ... | ,, 30 min. ... | ,, 60 min. |
| Spir. chol. ... ... | — ... | ,, 10 min. |

The vitality of the organisms was tested by inoculation in fluid media, and it was shown that the amount of antiseptic transferred did not interfere with the growth of the organisms employed.

### TANNO PUMILINE.

Arthur and Co., 69, Berners Street, send us a sample of the above. It consists of a saturated solution of digallic acid in the turpene of the essential oil of the pumilio pine effected by a process in use by the manufacturers. A compound is formed having the provisional formula $C_{36}\ H_{36}\ O_8$. The resulting substance as sent out is a dark sherry-coloured volatile fluid having a strong pumiline odour and leaving a thin micaceous layer on evaporation. The inventors claim that the fluid is particularly useful in cases where the epidermal layers of the skin are inflamed and where there is much itching, also in sweating feet and intertrigo. We have given it a fair clinical trial, more especially in the skin chafing conditions above mentioned. It acted well and sufficiently well for us to make further use of it in suitable cases. One case of excessive and foul perspiration of the feet was greatly relieved, the fœtor was removed and the perspiration certainly much lessened. It is decidedly a pleasant agent to use, and in no case has irritation been produced by it.

### DUROLINE, CARBOLINE.

Samples of these materials have been submitted by the New Wire-wove Roofing Company, 75a, Queen Victoria Street. The former is a semi-translucent, and the latter an opaque roofing material, both on a backing of wire gauze, rendering them tough and durable, but leaving them flexible and easily worked. Duroline is made in two qualities, and appears admirably adapted, from its cheapness and the ease with which it can be fixed, for forming a light roof over a temporary building, or a permanent roof over a factory or other building where glass is liable to fracture from vibration or expansion. Clear and simple instructions for fixing the material are supplied by the company, and should be carefully followed. Carboline is intended to take the place of felt or corrugated iron in roofs; its superiority over either consisting in its toughness. Both Duroline and Carboline appear highly inflammable, and should be used only where this quality is not a source of danger.

